# Severe eye complications from toxic epidermal necrolysis following initiation of Nevirapine based HAART regimen in a child with HIV infection: a case from Cameroon

**DOI:** 10.1186/s12887-018-1088-9

**Published:** 2018-03-13

**Authors:** Xavier Tchetnya, Calypse Asangbe Ngwasiri, Tiayah Munge, Leopold Ndemnge Aminde

**Affiliations:** 1 District Hospital Muyuka, Muyuka, Cameroon; 2 Faculty of Science, University of Buea, Buea, Cameroon; 3 Clinical Research Education, Networking & Consultancy (CRENC), Douala, Cameroon; 4 Regional Hospital Annex Buea, Buea, Cameroon; 5 School of Public Health, Faculty of Medicine, The University of Queensland, Brisbane, Australia; 6 Bamendjou District Hospital, Bamendjou, West Region Cameroon

**Keywords:** Toxic epidermal necrolysis, Nevirapine, Human immunodeficiency virus, Adverse drug reaction, Cameroon

## Abstract

**Background:**

Toxic epidermal necrolysis (TEN) is a rare life threatening dermatological disorder characterized by extensive epidermal detachment and erosion of mucous membranes. It is typically a side effect of some medications. Nevirapine, a nonnucleoside reverse transcriptase inhibitor (NNRTI) is one of the frequently used components of highly active antiretroviral therapy (HAART). Skin rash is its common adverse reaction, usually mild and rarely progressing to TEN. Ophthalmic involvement is common as well but rarely progresses to blindness especially in the pediatric population.

**Case presentation:**

We report the case of a 3 year 5 month old child diagnosed with HIV who developed TEN 8 days after starting a Nevirapine based HAART regimen. Drug withdrawal and supportive treatment alone were the modalities employed to achieve complete re-epithelization of lesions. Patient was lost to follow-up 6 months after being in care and was only seen 3 years later with total loss of vision.

**Conclusion:**

Blindness, though rare, can be a long-term complication of TEN in children especially with HIV infection. Physicians and patient caregivers should closely monitor these patients, especially during their early stages of treatment amongst others for development of adverse drug reactions. Long-term retention in care is pivotal for identification and prompt management of ocular and other chronic complications, albeit recognizing management challenges in low resourced settings.

## Background

Toxic epidermal necrolysis (TEN) or Lyell’s Syndrome is a rare idiosyncratic life-threatening severe cutaneous adverse reaction (SCAR) characterized by extensive detachment of the epidermis and erosion of mucous membranes [1]. It has the same pathogenesis as Steven-Johnsons syndrome (SJS), with the main difference being the proportion of body surface area (BSA) affected. When < 10% of BSA is affected it is classified as SJS, if > 30% is affected, it is considered TEN. An overlap syndrome is described when 10–30% of BSA is affected [1]. Mucocutaneous complications arise in about 90% of cases [2] and the ocular surface is one of the most commonly involved mucosal surfaces in TEN (50–67%) [3]. Survival from the often fatal acute stage of the disease is commonly followed by devastating ocular sequelae with bilateral blinding due to corneal scarring, and vascularization occurring in severe cases [3–5].

Compared with the general population, patients with Human Immunodeficiency Virus (HIV) have a 100-fold higher risk of developing TEN [6]. Drugs and upper respiratory tract infections constitute the main inciting agents especially in children. Sulfonamides, allopurinol, non-steroidal anti-inflammatory drugs (NSAIDs), antiepileptic and antiretroviral drugs (Nevirapine and Abacavir) are responsible for the majority of cases [7, 8]. Nevirapine, a dipyridodiazipinone is a non-nucleoside reverse transcriptase inhibitor (NNRTI) used to treat HIV − 1 infection. It binds directly to reverse transcriptase and blocks RNA- dependent and DNA-dependent DNA polymerase activity, causing disruption of the enzyme’s catalytic site [9]. Nevirapine based regimens of highly active antiretroviral therapy (HAART) have been widely used in resource-limited countries because of their efficacy, accessibility and comparative low cost [9, 10]. The World Health Organization (WHO) recommends Nevirapine and Efavirenz as first line drugs being part of an alternative protocol.

Amidst its wide availability and cost-effectiveness, its use is associated with serious toxicity with skin rash and hepatotoxicity being the most common adverse drug reactions (ADRs) observed. Skin rashes are usually mild and rarely progress to SJS or TEN (only about 0.5% of cases) [11]. Herein we report the case of a 3 year 5 month old child who developed TEN most likely due to nevirapine-based HAART regimen accompanied by bilateral blinding of the eyes.

## Case presentation

A 3 year 5 month old child was brought to our facility presenting with a five-week history of low grade fever associated with dry cough, watery rhinorrhea and progressive anorexia. These symptoms persisted despite treatment with paracetamol, quinine, cotrimoxazole and arthemeter/lumefantrine tablets, all taken as over the counter medications (OCM) on multiple occasions. His mother had died some months prior to this consultation, following a chronic illness she suffered for about 8 months. Physical examination revealed non-tender submandibular lymph nodes and splenomegaly. These findings led to the diagnosis of HIV infection (WHO stage III) with a CD4^+^ count of 311 c/μl. According to the Cameroon National AIDS Control Committee (NACC) guidelines (first line at the time; March 2013), he was initiated on a treatment regimen consisting Zidovudine (60 mg), Lamivudine (30 mg) and Nevirapine (100 mg) once daily. Nevirapine was started at a dose of 100 mg once daily for 2 weeks and a twice-daily increment was planned over the next 2 weeks. Cotrimoxazole 480 mg daily was also added as prophylaxis against opportunistic infections. Liver enzymes tested were within the normal limits. Eight days after commencing HAART, the patient presented systematically unwell with complaints of a three-day history of fever, restlessness, oral ulceration, sticky eyes and widespread painful pruritic skin eruptions.

**Fig. 1:**
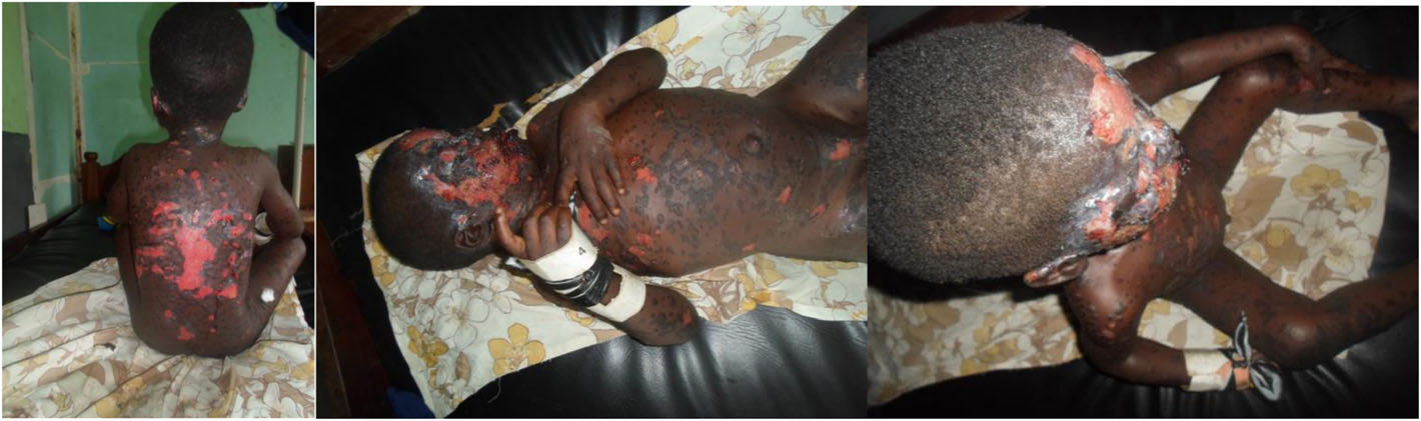
Massive facial and truncal epidermal loss with raw oozing dermis. Hemorrhagic crusting of the lips with targetoid lesions over the entire limbs

**Fig. 2:**
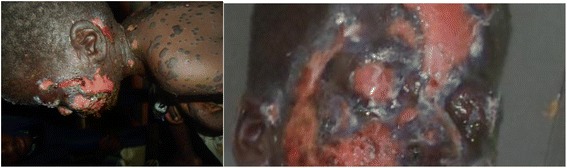
Marked conjunctival hyperemia with symblephara formation

His heart rate was regular (111 beats/min), axillary temperature was 37.2 °C and respiratory rate 37 breaths/min. Examination revealed massive facial and truncal epidermal loss with raw oozing dermis (Fig. 1). The epidermis featured irregular erythematous maculopapular zones, wide spread confluent bullae and targetoid lesions over the limbs. The blisters extended laterally on pressure (Asboe-Hansen sign). The entire skin covering the face, anterior parts of the trunk, denuded and peeled off with minor manipulation (Nicolsky sign). Hemorrhagic crusting of the lips was noted, with involvement of the skin over the genital region (Fig. 1). On ophthalmic examination, mood/affect was somnolent and external examination revealed extensive bullae (left eye), sloughing of the skin, with erythematous macules and patches (right eye). On anterior segment examination, there was marked conjunctiva hyperemia, palpebral synechiae and symblepharon formation (adhesion of the eyelids). (Fig. 2) No corneal opacification, ulcerations or perforation was noted. The iris, lens and fundus were not accessible for examination by a slit lamp.

At presentation to the hospital, the lesions had extended to involve about 90% TBSA using the modified Lund and Browder chart. A clinical diagnosis of TEN was made based on a temporal relationship, positive drug history, typical targetoid lesions (TBSA> 30%) and a positive Nicolsky sign. The absence of histopathological services made tissue diagnosis impossible. Available lab analyses revealed; white cell count at 8500 cells/mm3, hemoglobin level at 11.8 g/dl and blood sugar of 104 g/dl.

**Fig. 3:**
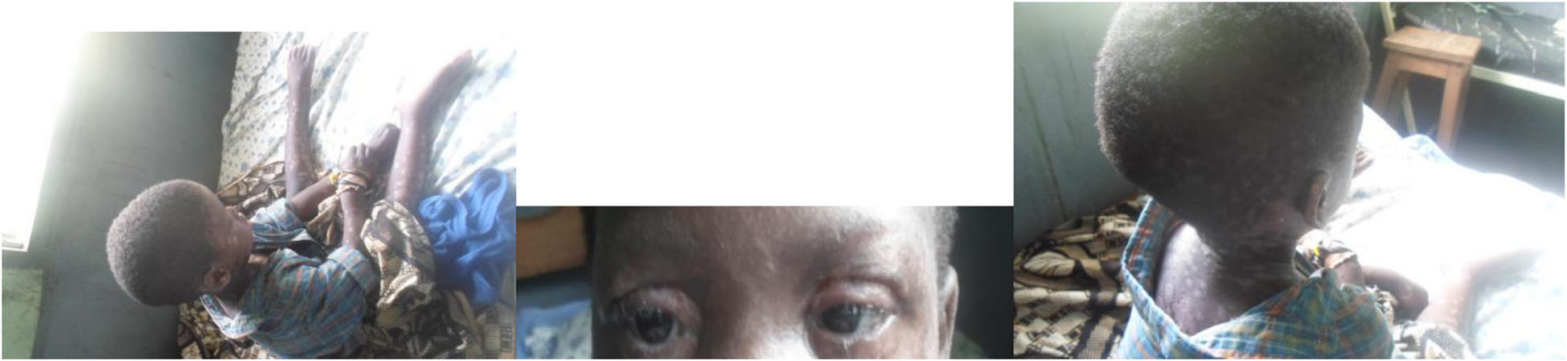
Resolution and re-epithelization of skin lesions

Following clinical suspicion, HAART regimen and cotrimoxazole were immediately stopped. With absence of a burn and intensive care unit in our facility, immediate management commenced in a restricted ward consisting of intravenous rehydration with ringer’s lactate and normal saline. Pain relief was achieved with intravenous paracetamol 500 mg 8 hourly whilst meticulous wound care was done with petroleum stained dressings. Ophthalmic opinion was sought and a combination eye drop of tobramycin 0.3%/ dexamethasone 0.1% was prescribed. The patient’s condition improved drastically over the ensuing 4–5 days, with resolution of skin lesions and reepithelization occurring 9 days after hospitalization (Fig. 3). However, supportive therapy continued until day 24 of hospitalization. A modified HAART regimen that included efavirenz instead of Nevirapine was started on day 17 whilst re-challenge with Cotrimoxazole was performed on day 21 of hospitalization with no development of any skin lesion. Fundoscopy done prior to discharge and during first follow-up visit (1 month later) was normal. There was no ocular complaint nor recurrence of rash during subsequent follow-up visits for 5 months. The patient was lost to follow up after being on treatment for 6 months and returned 3 years (October 2016) later with total loss of vision of both eyes. There was non-perception of light on visual field testing and further detailed ophthalmic examination was not done due to financial constraints.

The causality assessment using the Naranjo algorithm [12] revealed the ADR to be “probable” (Table 1). The fact that the patient had been taking Cotrimoxazole prior initiation of HAART, and re-challenged later without development of any lesions, made Nevirapine the most likely culprit.

**Table 1:** Naranjo Adverse Drug Reaction Probability Scale

Questions	Yes	No	DK
1. Are there previous *conclusive* reports on this reaction?	+ 1	–	–
2. Did the adverse event appear after the suspected drug was administered?	+2	–	–
3. Did the adverse reaction improve when the drug was discontinued or a specific antagonist was administered?	+ 1	–	–
4. Did the adverse event reappear when the drug was re-administered?	–	–	0
5. Are there alternative causes (other than the drug) that could on their own have caused the reaction?	-1	–	–
6. Did the reaction reappear when a placebo was given?	–	+ 1	–
7. Was the drug detected in blood (or other fluids) in concentrations known to be toxic?	–	0	–
8. Was the reaction more severe when the dose was increased or less severe when the dose was decreased?	–	–	0
9. Did the patient have a similar reaction to the same or similar drugs in *any* previous exposure?	–	0	–
10. Was the adverse event confirmed by any objective evidence?	+ 1	–	–

*DK* don’t know

The ADR is assigned to a probability category from the total score as follows: ***definite*** if overall score is 9 or greater, ***probable*** for a scores 5–8, ***possible*** for scores 1–4 and ***doubtful*** if the score is 0

## Discussion and conclusions

TEN is a rare complex immunological syndrome characterized by mucocutaneous blistering of skin and at least two mucous membranes; believed to share the same manifestation as SJS, differing only in severity [1]. About 0.4–1.5 cases per million population are reported worldwide with an approximate equal incidence in male and female children. The precise pathophysiology remains unclear but skin damage is thought to be either a delayed hypersensitivity reaction to certain medications or a response to epithelial cell antigens modified by drug exposure [13].

Sulphonamide antibiotics (38%) and nevirapine (20%) are reported to be the most common drugs causing TEN [14]. Nevirapine, a NNRTI constitutes an important part of HAART and is recommended by the WHO as one of the first line drugs, being part of an alternative protocol in the treatment of HIV infection. Amid serious toxicity associated with Nevirapine, it is widely recommended in low resourced countries like ours due to its availability, efficacy and comparative low cost.

Although our patient was not re-challenged with Nevirapine, the symptoms and signs were more consistent with Nevirapine induced TEN, so we believe that Nevirapine was responsible despite the patient being on cotrimoxazole, which is similarly a recognized inciting agent. In addition, the fact that the patient had received cotrimoxazole on multiple occasions as an OCM prior to HAART commencement and had a negative re-challenge to the drug made it a highly unlikely culprit. Symptoms developed about 8 days after starting HAART, further confirming findings of cohorts where TEN was shown to occur within 4–6 weeks of taking an inciting drug [15]. Our resource limited setting did not permit further assessment of liver enzymes whose gross elevation would have further accused NVP. Score of toxic epidermal necrolysis (SCORTEN scale, a stratification measure for assessing accurate prognostic indication was difficult to fully elicit in our patient due to lack of available laboratory results.

Once the culprit drug is removed, treatment is supportive. A study done in Malawi demonstrated this approach as an excellent stand-alone therapy [16], and our relative success here further illustrates this assessment. Fluids and analgesics are mandatory whilst antibiotics are beneficial.

Currently there is lack of consensus on any evidenced-based standard guidelines for the treatment of TEN and studies attempting to identify potential curative therapies like intravenous immunoglobulin and corticosteroids remain inconclusive [13].

A major challenge in resource-limited countries pertains to the limited available antiretroviral (ARV) agents particularly after first line agents have been exhausted or when serious toxicity occurs from an agent in one class. However, studies have shown a relative low (8–25%) risk of rash recurrence with EFV challenge [17, 18], justifying our use of EFV in this patient as recommended by the WHO. Four years ago, NVP was used as a preferred first line drug but current recommendations suggest Abacavir, Lamivudine and Efavirenz, with the use of NVP (and Zidovudine) only as alternative first line regimens.

Although recovery was near complete in our patient, he still ended up blind. The ocular surface is one of the most commonly involved mucosal surfaces in TEN (50–67%) [3]. Blinding ocular sequelae are the most devastating long-term consequences for survivors of an acute SJS/TEN. Patients who often survive the fatal acute stage commonly suffer devastating ocular sequelae that may develop many months after initial presentation, mandating the need for long-term follow-up of these children. Most complications are ocular surface abnormalities and not vision threatening as most children (90% of cases) maintain good vision long-term. Blindness as a complication is not common (5–9% of cases) [19].

About 27–80% of hospitalized patients with SJS/TEN develop acute ocular complications [20], with conjunctivitis (78%) being the most common clinical sign in the pediatric population [19]. Conjunctivitis together with conjunctival membrane and sub conjunctival hemorrhage constitutes one of the earliest signs. Other ocular complications in the acute phase of dermatological disease include conjunctival chemosis, corneal ulceration, corneal perforation, endophthalmitis and pseudo membrane formation [21]. Marked conjunctival hyperemia, sloughing and adhesion of the eyelids were present in our patient while corneal ulcerations, perforations or opacification (main causes of blindness) were absent.

Recently, aggressive intervention during the acute phase to limit damage of ocular surface and reduce incidence of long-term complications (which occurs in about 35% of patients) [4] has been the focus. Various treatment modalities have been employed, with preserved lubrication via corticosteroids most commonly used, as was the case with our patient. Amniotic membrane transplantation (AMT) would have been very beneficial in our case as it significantly improves visual acuity and ocular outcomes in patients with less severe ocular involvement, though insufficient to attenuate ocular inflammation in severe cases [22]. The low severity of our case as evidenced by no corneal involvement was an indication for AMT but financial restrictions and lack of appropriate social services made this unachievable. Fundoscopy done prior to discharge and during first follow up visit were normal. During subsequent follow-up visits, he had no eye complaint. Blindness noticed after 3 years of lost to follow up is a dismal prognosis. HIV positive patients have a high prevalence of dry eye syndrome and together with opportunistic infections of the eye like tuberculosis could have contributed to tear film instability and subsequently blinding in this patient. However, there was no evidence of tuberculosis infection.

We have presented the case of Nevirapine induced TEN in a 3 year 5 month old child who subsequently developed blindness. While our findings highlight some management challenges akin to low-income settings, it also emphasizes the need for strict monitoring by clinicians for patients on HAART especially during the early days. Furthermore, the case reiterates that chronic ocular complications following TEN can occur months to years after the initial presentation, warranting regular ophthalmologic check-ups and long-term lubrications as most will suffer dry eye syndrome. The value of retention in care cannot be overemphasized.

## Abbreviations

ADR: Adverse Drug Reaction
ARV: Antiretroviral Drugs
BSA: Body Surface Area
DNA: Deoxyribonucleic Acid
EFV: Effavirenz
HAART: Highly Active Antiretroviral Therapy
HIV: Human Immuno-Deficiency Virus
NACC: National AIDS Control Committee
NNRTI: Non-Nucleoside Reverse Transcriptase Inhibitor
NSAIDs: Non-Steroidal Anti-Inflammatory Drugs
NVP: Nevirapine
OCM: Over the Counter Medication
RNA: Ribonucleic Acid
SCAR: Severe cutaneous adverse reaction
SCORTEN: Score of Toxic Epidermal Necrolysis
SJS: Steven-Johnson Syndrome
TEN: Toxic Epidermal Necrolysis
